# Disclosure of HIV status among Shan female migrant workers living with HIV in Northern Thailand: A qualitative study

**DOI:** 10.1371/journal.pone.0216382

**Published:** 2019-05-02

**Authors:** Arratee Ayuttacorn, Arunrat Tangmunkongvorakul, Patou Masika Musumari, Kriengkrai Srithanaviboonchai, Amporn Jirattikorn, Linda Aurpibul

**Affiliations:** 1 Faculty of Social Sciences, Chiang Mai University, Chiang Mai, Thailand; 2 Research Institute for Health Sciences, Chiang Mai University, Chiang Mai, Thailand; 3 Department of Global Health and Socio-epidemiology, Kyoto University School of Public Health, Kyoto, Japan; 4 Japan Foundation for AIDS Prevention, Tokyo, Japan; 5 Faculty of Medicine, Chiang Mai University, Chiang Mai, Thailand; International Medical University, MALAYSIA

## Abstract

**Background:**

Disclosure of HIV status is a critical gateway to HIV prevention. Despite many studies on this topic, there is a gap in knowledge regarding HIV status disclosure and risky sexual behavior in HIV-infected female migrant workers. The current study addressed this research gap, and focused on HIV-infected Shan female migrant workers in Northern Thailand.

**Methods:**

This study conducted in-depth interviews with 18 HIV-infected Shan female migrants (aged between 23–54 years old) and 29 healthcare workers in district hospitals in Chiang Mai. Content analysis was employed to identify particular themes related to HIV status disclosure, sexual risk behavior, and ART adherence.

**Results:**

We found that non-disclosure to husbands/partners was mostly related to fear of marital conflict and of losing social and financial support. Non-disclosure prevented Shan female migrant workers from negotiating condom use with their partners. Reasons for not disclosing to friends, family and other community members were mostly related to feared rejection and discrimination due to HIV-related stigma. Accounts of condomless sex in the context of HIV status disclosure suggest that gender norms and male dominance over women influenced decision-making for safe sex. Lastly, some female migrant workers perceived low risk of HIV transmission with good adherence to the ART.

**Conclusions:**

This study highlighted the complex challenges of HIV status disclosure among HIV-positive Shan female migrant workers and the link between disclosure/non-disclosure and condom use. There is a pressing need to create realistic disclosure mechanisms that take into account the socio-cultural barriers to disclosure including marital conflicts, stigma, and gender norms. Messages to encourage condom use should be delivered carefully so that knowledge of the HIV transmission reduction qualities of good ART adherence does not serve as a barrier to condom use.

## Introduction

Thailand has been a transit and destination country for migrant workers from neighboring countries since the early 1990s. It is estimated that 3.6 million migrants are living in Thailand [1]. The majority (2.7 million) of migrant workers are from Myanmar (86%), Cambodia (10%), and Laos (4%). Female migrant workers represent approximately 45% of all low-skilled migrant workers. In addition, 1.6 million migrant workers have irregular status [2]. Of the 2.3 million migrants from Myanmar, the Shan people represent the second largest ethnic group behind the Burman ethnic group [3]. In Northern Thailand, however, the Shan are the largest ethnic group, with an estimated population of 200,000 who live and work in Chiang Mai [4]. More than 90% of migrants work in low-paid and high-risk jobs in the service sector, agriculture, and construction industries [5, 6], and almost half of sex workers in northern Thailand were Shan migrants [7].

In 2017, the estimated prevalence of adult HIV in Thailand was 1.1%. The epidemic was concentrated among men who have sex with men, sex workers, transgender people, people who inject drugs, prisoners, and migrants [8, 9]. Approximately 9% of the people who live with HIV in Asia and the Pacific, reside in Thailand [10]. Northern Thailand, a destination for migrant workers, has had the highest HIV prevalence in the country, and is the setting for this study [11, 12, 13]. There is little research on HIV prevalence among migrant workers in this region. The latest study in 1999 showed that the rate of HIV-1 prevalence among migrant workers was 4.9% (5.7% among men and 3.8% among women) [14]. Previous research has documented a myriad of factors leading to the vulnerability of migrant workers to HIV. Risks related to national policy, socio-cultural context, and sexual risk behaviors [15, 16, 17, 18]. These included limited knowledge about HIV/AIDS, low HIV risk-perception, lack of awareness and/or limited access to migrant-friendly sexual and reproductive health, and limited or inconsistent condom use [19, 20, 21, 22, 23, 24, 25, 26].

The increasing number of migrant workers living with HIV in Thailand has set in motion programmatic efforts, which mostly have focused on improving access to antiretroviral treatment (ART) in this population [27]. Since 2001, registered migrant workers in Thailand are eligible to access ART through the Thai health care insurance system, obtained either through the social security scheme or the migrant health insurance system [3, 27]. In 2013, the Thai government, through its Ministry of Public Health, expanded its universal health care policy allowing both documented and undocumented migrant workers to purchase health insurance covering ART provision [28].

Little research has been conducted to guide programmatic efforts on issues related to disclosure of HIV positive status or the sexual and reproductive life of HIV-infected migrant workers in Thailand. Disclosure of HIV positive status is a critical step in the management of HIV infection. Previous studies on HIV disclosure to partners, family members, and other network members have shown that it is a double-edged sword. Disclosure has been linked to increased HIV testing in sexual partners, decreased risky sexual behaviors, increased social support, and increased adherence to ART [29, 30, 31, 32, 33]. On the other hand, disclosure of HIV has also been associated with a range of adverse outcomes in both HIV-infected males and females. Due to socio-cultural and gender norms that exist in many settings, females living with HIV are particularly likely to experience blame, discrimination, rejection, loss of financial support, marital conflicts, as well as physical and emotional violence following disclosure of HIV status [34, 35, 36, 37].

Non-disclosure of HIV-positive status, specifically in the context of sexual partnerships, can lead to HIV transmission in sero-discordant couples if appropriate prevention strategies are not adopted [36, 38]. Thailand’s public health policy and NGOs recommend disclosure as a way to prevent HIV transmission [39, 40]. The National Operational Plan Accelerating Ending AIDS’s framework instructs healthcare providers to counsel and facilitate disclosure to the partner, family and community [9, 41]. Disclosure by HIV-infected female migrant workers might particularly be challenging with respect to many migrant-specific issues such as illegal status, lack of health insurance, as well as financial and job insecurity. There is, however, a paucity of research examining disclosure in HIV-infected female migrant populations.

Chiang Mai is the largest province in Northern Thailand both in geographical area and population size. It is the economic hub of Northern Thailand, borders Myanmar, and attracts a growing number of migrant workers from the neighboring country [2, 25]. Therefore, this study examines HIV status disclosure and the sex behaviors of HIV-infected Shan female migrant workers in Chiang Mai. In particular, reasons for non-disclosure and socio-cultural and contextual influences on HIV status disclosure in this population were explored.

## Methodology

This study presents the results of the qualitative arm of a mixed-methods study that aimed to investigate health behaviors, quality of life and use of health services of migrant workers living with HIV in Chiang Mai. The participants in the qualitative study were recruited among those who participated in the quantitative survey. Briefly, the quantitative survey was conducted between November 2016 and April 2017 in 12 community hospitals in Chiang Mai that had the largest number of HIV patients. In total, 333 (121 males and 212 females) HIV-infected migrants participated. The qualitative study was conducted on the same day as the quantitative study. Participants interested in joining the qualitative study were provided with information regarding the study objectives. Participants in the qualitative study were purposively selected to cover variation in sex, age and length of HIV infection. Three to four participants were recruited from each hospital.

### Data collection

Data were collected through in-depth interviews aided by a semi-structured interview guide. The interview guide was developed through the objectives of the study to learn about the way of life of migrant workers living with HIV, and to have more detailed information in some questions asking in the questionnaire. The questions in the interview guide were validated by discussions among research team members who were qualitative experts and have had experiences in doing research with migrants and people living with HIV.

The in-depth interviews continued until the data saturation was reached when replication of data occurred, and the data were suitable to present interesting findings. Eventually, 43 migrant workers (21 males and 22 females) participated in the qualitative study. This current paper particularly focuses on data from 18 HIV-infected female Shan migrants (4 female migrants were excluded because they were from other hill-tribe ethnic groups). The qualitative research also included a key informant group of healthcare providers who worked in HIV clinics. The healthcare providers in this study were clinic nurses and health volunteers who provided health services to HIV-infected patients, especially among migrants, in out-patient departments. We invited 2–3 healthcare providers from each hospital (12 hospitals) to be interviewed. In total, 29 healthcare providers participated in the qualitative arm of the study.

The in-depth interviews were conducted by trained interviewers, and took place in private rooms at the hospitals to ensure participants’ privacy. The interview guide consisted of items on personal and family background, self-care, ARV adherence, sexual behavior, HIV disclosure, and relationships with partner, family, friends, and community. The interviews were conducted mostly in Thai or the Northern Thai dialect since most participants could communicate in the Thai language. However, participants were allowed to speak in Shan if they preferred. Three research team members were fluent in Shan, and could communicate well with the participants. The interviews lasted between 45 to 90 minutes depending on the amount of information given by the participants. The field notes were made during the interviews to record some essential details, such as participants’ emotions, researcher’s thoughts, and initial findings. All in-depth interviews were digitally audio-recorded.

### Ethics statement

The study received ethical approval from the Human Experimentation Committee at the Research Institute for Health Sciences, Chiang Mai University (Certificate of Ethical Clearance No. 17/ 2516). Participants were informed that the interviews would be audio-recorded, and all provided written informed consent. The interviews were conducted in a place where participants felt safe and comfortable. We used index numbers to identify participants’ instead of real names to preserve confidentiality.

### Data analysis

Audio recordings of interviews were transcribed verbatim in Thai. All transcripts were not returned to participants for comments, but they were validated through research team dialogue and discussions. In two cases, the interviews were translated and transcribed from Shan into Thai by a Thai research team member who could speak Shan. The transcripts and field notes constituted the final material for analysis. The data were analyzed using content analysis [42]. This method consists of a systematic coding and categorizing textual information to analyze data qualitatively and quantify the data at the same time [43]. ATLAS.ti software (version 8.0) was used in the coding process, and an initial coding scheme was developed in line with the topics outlined in the in-depth interview guide.

The research team employed an inductive process, and the relevant codes were sorted into sub-categories. These emergent sub-categories were used to organize and group the data into the categories. Three researchers discussed and reflected to revise tentative categories. Finally, the categories were formulated into the themes (such as sexual life and vulnerability to HIV infection, barriers of HIV status disclosure, stigmatization, and ART adherence). Passages most relevant to the study were later translated into English and presented in the current paper. This study also applied triangulation [44] by combining different approaches including comparing data from both the questionnaires and in-depth interviews. We interviewed both patients and health staff to further cross check the data. In addition, the data were validated and sorted through a process of discussions among the research team which formed a consensus on the research findings. This method is appropriate for investigating sexual behavior, perception, and HIV disclosure status which are all sensitive issues. Socio-demographic data from the quantitative questionnaire were used to provide background information of participants.

## Results

### Socio-demographic characteristics

The participants ranged in age between 23 and 54 years (mean age 38.6). They were all Buddhists and all born in Myanmar. Almost all, thirteen, reported having never attended school. The majority (83.3%) were employed as laborers on construction sites or farms, or as housekeeping staff in a private home or a shop/office/company. Their income was quite low as all of them earned less than 10,000 Baht (294 USD) per month, with half only earning 5,000 Baht (147 USD) per month. Most have been in Thailand for more than 10 years (mean = 16.0 years). While all had an official Thai identification card for foreigners and health insurance, only 12 had a legal work permit. Thirteen were married or lived with their spouse, and 5 were separated, divorced or widowed. Of those living with a spouse, only 3 had HIV positive partners. The remaining partners were HIV negative or of unknown status. All but 2 lived with family (spouse, family member, etc.) and half said their combined family income was insufficient (see Table 1).

**Table 1:** Socio-demographic background of female Shan living with HIV (N = 18).

Socio-demographic background	Frequencies
Age in years: (Range 23–54); Mean (SD) = 38.6 (8.8)	
Place of Birth: Myanmar	18 (100%)
Education: Never attended school	13 (72.2%)
Religion: Buddhism	18 (100%)
Job: Employed	15 (83.3%)
Monthly income (n = 15): Less than 10,000 Baht	15 (100%)
Time in Thailand (years): Mean (SD) = 16.0 (7.3) More than 10 years	14 (77.8%)
Have official Thai ID Card for foreigners	18 (100%)
Have a work permit	12 (66.7%)
Have health insurance	18 (100%)
Marital status: Married/Living with spouse	13 (72.2%)
HIV status of partner (n = 13): Negative or Unknown	10 (76.9%)
Number of family members: 2 or more	16 (88.9%)
Family financial status: Insufficient	9 (50.0%)

All participants were sexually active. The median age at first sex was 19 years old, and most participants have had 2 or more lifetime sexual partners. The majority (88.9%) remained sexually active after learning their HIV-positive status, and reported having sexual intercourse 1–3 times or more per month. Many (43.8%) reported that their last sexual encounter occurred within a week of study participation. Most (93.8%) of the sexual partners were either their husbands or other regular partners (see Table 2).

**Table 2:** Sexual behavior of female Shan living with HIV (N = 18).

Sexual behavior	Frequencies
Have ever had sex	18 (100%)
Age at first sex: Median = 19	
Number of lifetime sexual partners: 2 or more	16 (88.9%)
Still having sex after knowing HIV status	16 (88.9%)
Frequency of having sex in the past year: 1–3 times per month (n = 16)	12 (75.0%)
Last time had sex (n = 16): 1–7 days ago	7 (43.8%)
Sexual partner at last time (n = 16): Husband or Regular partner	15 (93.8%)

Among female Shan migrant workers living with HIV, the median time since HIV diagnosis was 5 years. Half were tested for HIV because of pregnancy (50%). Most (66.7%) reported getting HIV from sexual partners. The majority (83.3%) have been on ART for at least one year, and reported excellent adherence to their medication. Only half disclosed their HIV status to their sexual partners, and very few revealed this information to family or to others in the community (see Table 3).

**Table 3:** HIV information of female Shan living with HIV (N = 18).

Information on HIV	Frequencies
Years since HIV diagnosed: Median = 5	
0–1 year	1 (5.6%)
2–5 years	9 (50.0%)
6–10 years	4 (22.2%)
More than 10 years	4 (22.2%)
Main reason to get tested for HIV	
Physical exam (i.e. for work permit)	4 (22.2%)
Illnesses	5 (27.8%)
Pregnancy	9 (50.0%)
Who do you think transmitted HIV to you?	
Sexual partner	12 (66.7%)
Don’t know / Not sure	6 (33.3%)
Began ART	
Less than 1 year	3 (16.7%)
1 year or more	15 (83.3%)
Adherence to ART (self-report)	
Excellent (95–100%)	12 (66.7%)
Good (80–94%)	5 (27.8%)
Fair (50–79%)	1 (5.5%)
Apart from healthcare providers, who knows your HIV status?	
Husband / Sex partner (n = 18)	9 (50.0%)
Family member(s) (n = 18)	3 (16.7%)
Other people in the community (n = 18)	2 (11.1%)

The healthcare providers who participated in the in-depth interviews were aged between 26–55 years, and the majority were female and had finished their education to Bachelor degree level. Almost all of the participants were nurses. All had provided services to HIV infected patients in the clinics for at least 5 years, and 23 of them had provided services to HIV infected patients for 10 years or more (see Table 4).

**Table 4:** Socio-demographic and professional characteristics of healthcare providers (N = 29).

Professional characteristics	Frequencies
Age in years: Range 26–55	
Sex: Female	25 (86.2%)
Education: Bachelor degree	24 (82.8%)
Career	
Nurse	24 (82.8%)
Nurse assistant/Health volunteers Length of time providing services to HIV patients: 10 years or more	5 (17.2%) 23 (79.3%)

The themes that emerged from the study are presented below. The quotes, presented in support of the themes, were slightly edited for ease of understanding.

#### Sexual life and vulnerability to HIV infection

Multiple factors shaped the vulnerability of Shan female migrant workers to HIV infection. Shan female migrants in this study were all sexually active and reported frequent changes in sexual partners. While some Shan female migrants arrived in Chiang Mai with their husband/partner, others arrived alone, either because they were single or because they left their family and husband behind in their home country. Reasons for the frequent change in partners were the death of a husband/partner, separation, and the need to find a new partner. Most Shan female migrants thought that they were infected with HIV by their former husband/regular partner. Being HIV positive, however, was not a barrier to engaging in a new relationship.

*I migrated to Thailand with my husband and three kids*. *My first husband passed away 7–8 years ago from pneumonia*. *After my husband died I raised three kids and then I got a second husband*. *He had many wives before we met*, *after we lived together for 3 years*. *I didn’t know that he was a man of loose morals; he took another woman into our house*. *Later I broke up with him and found a third husband*. *I think I got HIV from the second one*.

(P027, 43 years old, diagnosed with HIV for less than 1 year)

Health staff also pointed out that Shan female migrants often change their partners and initiate unsafe sexual behaviors leading to HIV infection.

*Many of the migrants got married in Burma*. *When they moved to Chiang Mai they had new partners again*. *Some had 2–3 people; after a few months they broke up*. *When they have sex with new partners*, *they are not aware about HIV protection*.

(S015, clinic nurse)

### HIV status disclosure

#### Public health policy and HIV positive status disclosure

Health care providers follow the Thai’s National Operational Plan Accelerating Ending AIDS which recommends HIV status disclosure to partners, family, and the community. Therefore, they systematically encouraged Shan female migrants to disclose, and when needed, they offered their assistance to facilitate the process of disclosure. One clinic nurse reported the following:

*We ask the patient to inform their family about their test result*. *If they do not want to let them know*, *I ask the reason why*. *If they tell me that they are not brave enough to tell their partner*, *I offer to make an appointment and explain the test result to their partner*.

(S020, clinic nurse)

#### Difficulty telling a new husband/regular partner their HIV positive status

Only half of the Shan female migrants said that their husband/regular partner knew about their HIV positive status. In most cases, their husbands/partners were either HIV negative or of unknown status. Non-disclosure of HIV status posed a serious barrier to HIV prevention by impeding safe sex practice for the couple and HIV testing for husband/partner. The following section examines reasons for non-disclosure and its impact on condom use in the context of migration.

#### The need for financial and relational security

The Shan female migrants in this study faced enormous financial burdens caring for themselves and their children with notably low monthly incomes. They largely depended on their partners for financial, social, and relational security. Therefore, they feared that disclosure of their HIV status could lead to marital conflicts, with the potential risk of losing their husband/partner. This could lead to the loss of social and financial support. The situation is illustrated in the following quotes:

*I got pregnant with my third husband*. *I knew about my HIV positive status 3–4 months ago when I saw the doctor while pregnant*. *My third husband is HIV negative*. *He is Thai from Fang district; we worked at the same place when I was with my second husband*. *After we broke up*, *I was alone; he saw that I worked hard and felt sorry for me*. *Then we fell in love and began living together*. *Now he runs his own business*, *doing small construction projects such as tiling work*. *After we got married*, *I stayed with him*, *and he documents me as his employee*.

(P027, 43 years old, diagnosed with HIV for less than 1 year)

Another Shan migrant received financial support from a new husband, she stopped working and became a housewife. She got pregnant although she perceived the risk of HIV transmission to her husband.

*My second husband is Shan*, *we worked together on a construction site*. *After six years of marriage he passed away and I think I got HIV from him while we were married*. *I had no place to live*, *so my friend suggested that I work in a karaoke shop*. *I needed money to support my father and a kid*. *I met my third husband*, *a Thai in the karaoke shop*. *After he proposed to me*, *I stopped working*, *became a housewife and had a [new] kid*. *Sometimes I earn extra money by selling the baskets that I weave*, *but my husband prefers that I don’t work*, *and instead just look after the household and take care of our child*.

(P033, 38 years old, 3 years since diagnosed with HIV)

The stories were confirmed by healthcare providers. HIV infected migrants would not tell their partners about their HIV status to avoid conflicts and the risk of abandonment since they needed social and financial support from the partners.

*Shan migrants need support; some live in Thailand for a long time and later their husbands pass away*. *It is the nature of humans to seek love*. *I ask many women why they did not tell their new partners [about their HIV status]*. *They said they could not take the risk*. *If their new partners are Thai*, *they will have a place to live forever*.

(S019, clinic nurse)

Some participants continued having children although in general, health care providers discouraged it given the risk of HIV transmission to the partner (if HIV-negative) or to the baby. Having children was a way of consolidating one’s relationship with their husband and provided a greater sense of relational security to the female migrant workers. However, in other instances, the need for a child emerged from the husband. It is illustrated in following quotes:

*My first husband passed way [from AIDS] and I had one daughter with him*. *Later I met a new husband; he was already divorced from his wife*. *I quit from my job after I got married to my second husband*. *He had kids with his ex-wife*, *but all of them were girls; he wanted to have more kids with me*. *He has never used condom*, *eventually I got pregnant and we had a boy*.

(P033, 38 years old, 3 years since diagnosed with HIV)

Healthcare providers in HIV clinics also provided their views regarding HIV-infected female migrants’ intentions to get pregnant. Some said that even though Shan migrant woman often know that their new husbands are HIV negative, they do not employ birth control and eventually have a new child.

*She has an HIV negative husband*. *She has one kid already [with an ex-husband]*. *She actually did not want to have a second kid*, *but her husband wanted to*, *so she did not use birth control*. *She hadn’t been taking ARV for very long*, *and then got pregnant*. *We cannot forbid those living with HIV [to have another kid]*, *but we keep telling them that if they want a kid*, *please consult with us first*.

(S020, clinic nurse)

*When she got pregnant with her first kid [with the first husband]*, *she knew she was HIV positive*. *She did not take ARV because it was so expensive*. *She did not have access to public health insurance since she did not have any legal documents*. *Later an NGO helped her to get the insurance*. *She joined the treatment program before she got pregnant with the second kid*. *She did not use birth control*, *and then she had the second kid*, *while her new husband was HIV negative*. *However*, *she takes ARV drugs effectively and the second kid is HIV negative*.

(S029, health volunteer)

#### Non-disclosure, a barrier to condom use

Shan female migrants, particularly those who did not disclose HIV status, had difficulties to negotiate condom use with their partners because they feared doing so could raise suspicions regarding their HIV positive status.

*I got infected from my first husband; we have a kid together*. *After he passed away*, *I got a new husband who is Shan*. *I got pregnant with the second kid and the doctor told me that I was HIV positive*. *Since then I take ART everyday*. *But I’m afraid to tell my husband about my HIV status*. *He wouldn’t accept it*. *I’m not sure if he would beat me or not*. *I never ask him to use condoms even though I know that I could pass HIV to him*.

(P043, 35 years old, 5 years since diagnosed with HIV)

In rare instances, female migrant workers were resolute in using condoms and made up reasons to justify their condom use. In the following quote, the participant faked having cervical cancer so as to negotiate condom use with her partner.

*I had a second husband when I moved to Chiang Mai*, *He passed away 10 years ago*. *I got sick and the doctor suggested that I have a blood test*. *I think my second husband infected me with HIV*, *but he was never tested*. *I have been taking ART for 12 years*. *My new partner doesn’t know that I am HIV positive*. *I ask him to use condoms and lie that I have cervical cancer*. *I feel terrible that I cannot tell anyone*. *I think that they would not accept this and reject me*, *even though no one has said so*. *I feel really sorry for myself*.

(P006, 54 years old, 12 years since diagnosed with HIV)

In this case, a Shan female migrant did not disclose her HIV status and isolated herself from others. She internalized the stigma of society’s negative views of HIV infection. Her own self-stigmatization made her especially isolated and vulnerable.

#### Unsafe sex despite disclosure of positive HIV status

This study also revealed that the decision to use condoms largely depended on the male partner. Some female migrant workers reported that their husbands ignored their demands to use condoms, although they were aware of their HIV status. This illustrates the role of gender power in safe sex practice,

*I do not use condoms regularly*, *my husband tells me that it does not matter*, *everyone has to die*. *The person who does not have HIV may die before the infected one*. *I told him to find a new wife who is not infected*. *He does not want to*, *he said we have 2 kids together and we will stay together even though we are poor*.

(P011, 23 years old, 2 years since diagnosed with HIV)

Another Shan female migrant revealed that her husband was informed about her HIV status but he still refused to use a condom.

*Doctors told us to use condoms*, *but my husband said it will not transmit since we have lived together for many years*. *He refuses to use a condom*. *I told him to not have sex with other women because he could infect me with some other disease*.

(P033, 38 years old, 4 years since diagnosed with HIV)

#### HIV stigma among family members and the community

Health care providers, as part of the national HIV policy, encourage HIV-infected people to disclose their HIV status in the community and other social network members. It is assumed that this might benefit HIV-infected individuals because of the potential social support they might receive from the network members following disclosure. Shan female migrants, however, have indicated their reluctance to disclose their HIV status to either friends, family, and the community. Fear of being stigmatised, marginalized, or of losing one’s job was often invoked as the reasons for not disclosing their status. HIV infection was still associated with sexual promiscuity. These views are demonstrated by this quote:

*I can’t tell my friends*. *It is so disgraceful in Shan society*. *They would look at me like an immoral woman who can’t do any good because I am promiscuous*. *They do not even consider that I got this infection from the husband*.

(P003, 39 years old, 5 years since diagnosed with HIV)

In a few instances, fear of disclosure was based on firsthand experience of witnessing their friends’ gossipping or negative attitudes toward HIV or people infected with HIV. This leads Shan female migrants to avoid taking the risk of stigmatization.

*I have not told my kids or my friends*. *Only my husband knows about my HIV status*. *If the others knew about this*, *they would look down on me and be disgusted with me*. *I have seen this before with another HIV positive woman; no one ate the food she cooked*, *and finally she died*. *I’m afraid to be treated like this*, *so I won’t tell my husband’s parents although we live together*.

(P031, 47 years old, 4 years since diagnosed with HIV)

Multiple strategies were employed to avoid disclosure in the community. These included chosing to refill ART medication from a hospital far from their community, not seeking care from HIV clinics, and making appointments with health care providers on days that are not specifically reserved for HIV patients.

*I stay with my second husband’s family in Mae Wang*. *My husband is Thai and he is HIV positive*, *we haven’t told his family and other people about HIV status*. *I went to Mae Wang Hospital and I did not feel satisfy to go there*. *Then my husband took me to Sanpahthong hospital which is far from my village*. *Even though my insurance card is from Mae Wang but I could still receive health services from San-pah-thong hospital*. *The staffs here are so friendly and treat me well*.

(P031, 47 years old, 4 years of HIV diagnosed)

In addition, participants who had not disclosed their HIV status to their employers had to make up reasons to justify their absence from work when they went for an ART refill.

*Many migrants do not tell anyone because they are afraid they might be fired from their jobs*. *For some patients*, *after they move and are far from this hospital*, *they are not able to get their ARV drugs regularly because they didn’t have enough money for transportation*. *We offered to refer them to a nearby hospital*, *but they declined our offer*. *They are concerned there will be neighbors at the nearby hospital that would gossip about their HIV status in local community*.

(S004, clinic nurse)

#### ART adherence

Most participants displayed a great sense of responsibility and reported that they followed recommendations from health care providers to take HIV medication on time and everyday, despite experiencing treatment side effects. They mentioned that the health care providers emphasized the importance of good adherence to avoid treatment failure and drug resistance. They also received advice for good nutrition and physical exercise. The improvement of health status after starting ART was a motivating factor to maintain good adherence to the medication and to regularly attend medical visits.

*I have been treated with ARV for 12 years*. *I feel good and strong*. *I take my ARV at 8 am and 8 pm every day*. *The doctor told me to take drug consistently*.

(P006, 54 years old, 12 years since diagnosed with HIV)

Most of the Shan female migrants had adhered to ARV, however healthcare staff noted that some migrants often moved their workplaces and could not continue ART treatment in the same hospitals.

*They take care of themselves well and come to the HIV clinic regularly*. *Even though they are migrants*, *they take their ARV drugs every day*. *There are not many problems with drug resistance*. *Some come with family who could remind them to take the drugs*. *However*, *the problem of migrants is that they always move from place to place for work*. *It is difficult for us if they are lost to follow up*.

(S012, clinic nurse)

The desire to be healthy and to take care of their children emerged as an important factor that motivated female migrant workers to adhere to HIV medication and recommendations from health care providers. Most Shan female migrants felt that their children depended on them, and because they were in a foreign country, they could not rely on family members or relatives to help with care and support for their children.

*I got pregnant with my second kid and I was told that I was HIV positive at that time*. *I had ART for a while and then I stopped the treatment for many years*. *I just started a new treatment this year because I want to live longer*. *My kids are so young*, *if I die*, *no one would take care of them*. *I have to tolerate the ART side effects*. *I had an allergic reaction and dizziness*, *then the doctor changed drugs for me*. *Now I take ART every day and see the doctor every 3 months*.

(P039, 53 years old, 5 years since diagnosed with HIV)

Frequent change of jobs and residence were cited as barriers to ART medication adherence. In addition, there was interplay between fear of disclosure, financial insecurity, and ART adherence. Some Shan migrants preferred refilling ART in health facilities located far from the community to avoid inadvertent disclosure.

#### Perceived risk of HIV transmission and risky sexual behavior

Participants received education on both HIV prevention and treatment from health facilities. While they were encouraged to systematically use condoms during sexual intercourse and to avoid getting pregnant, the message that good adherence to ART substantially reduces the risk of both sexual and mother-to-child transmission of HIV has lowered their perceived risk of transmission.

*I started taking ARV when I was 4 months pregnant*. *My second kid was HIV negative*, *so I knew that I could have a kid without transmitting HIV*. *The doctor told me that as long as I take the ARV*, *my kid will not be infected with HIV*. *I learned from their advice*.

(P011, 23 years old, 2 years since diagnosed with HIV)

Healthcare staff confirmed that they gave knowledge to the patients about good ART adherence that could reduce HIV transmission.

*Migrants learned about HIV transmission issues from the health staff*. *HIV will not possibly transmit to the partners as long as they take their ARV drugs regularly*. *Therefore*, *they may not use condoms as far as their viral load is low*.

(S007, clinic nurse)

*I told my patients that the risk of infection is near zero when they are on ART regularly*. *Their viral load will be very low*. *And for those who are pregnant*, *the virus will not infect the fetus*.

(S006, clinic nurse)

## Discussion

This study provides insight into the factors that shape disclosure of HIV positive status and sexual behavior of Shan female migrants living in Chiang Mai. Our study has highlighted the vulnerability of female migrant workers to HIV acquisition, and the potential risk of HIV transmission to other partners in the context of gender and financial power, and socio-cultural norms. The concealment of HIV status to their partners and communities is also related with level of stigma and discrimination against people with HIV. Moreover, some Shan female migrants in the study had good knowledge regarding HIV prevention and treatment; they perceived that good adherence to ARV drugs could reduce the risk of HIV transmission to their sexual partners.

Although it is not uncommon for Shan female migrant workers to come to Thailand while they are still single, many were already married prior to migration. Many arrived in Chiang Mai with their husband/partner. Most Shan female migrants in our study were employed (officially or informally), however, their income was not sufficient to meet their financial demands. Since the majority of our participants earned a monthly income of 10,000 Baht (294 USD) or less, they largely relied on their husband/partner as a source of financial and social support. Against this backdrop of social and financial insecurity, we found that many female migrant workers were at least in their second or third marriage/partnership, following either the passing away of, or separation from, their previous husband(s)/partner(s). Most of them learned they were infected with HIV when they became pregnant (tested for HIV during antenatal care visits) or when they developed disease symptoms; suggesting a gap in the delivery of HIV testing services for migrant population in Thailand.

Previous studies have associated disclosure of HIV status with increased social support from partners, family and friends [29, 30, 31]. Disclosure is also a critical gateway to HIV prevention, because it can be a strategy for negotiating safe sex with their husband/partner and it can encourage HIV testing among sexual partners [45]. The findings in the present study contradicted previous studies, it documented that Shan female migrant workers were not only encouraged to disclose their HIV status, but they also received education regarding HIV prevention and treatment. The message emphasized systematic use of condoms as a way to prevent transmission, and the importance of adherence to ART to ensure treatment success and avoid drug resistance. However Shan female migrant workers did not disclose their HIV status to their husband/partner upon becoming aware of their HIV positive status. This has serious implications for the prevention of HIV since most of their husbands were either HIV-negative or of unknown HIV status. Non-disclosure to friends, family, and other community members indicates the level of stigma present in the Shan community.

Disclosure of HIV is a critical process that sometimes results in negative reactions, and ultimately, loss of social support from the social network particularly in settings where HIV is still highly stigmatized and attached to sexual promiscuity [34, 37, 38, 46]. Our study reiterated the previous studies findings that stigmatization is the main factors for HIV non-disclosure. Fear of rejection, of being stigmatized, the uncertainty of the impact on the relationship with husband/partner, and the social stigma associating HIV with promiscuous sexual life tipped the balance for not disclosing positive HIV status. Consequently, some female migrant workers did not negotiate condom use with their husbands/partners fearing that this could lead to inadvertent disclosure of their status. However, Shan female migrants had relatively good knowledge regarding HIV prevention; this is in contrast with previously made assumptions that Shan female migrants do not encourage condom use because of their limited knowledge regarding HIV prevention [7, 19, 23].

Consistent with previous research [47, 48, 49], this study also revealed that unsafe sex was still common even in the context of HIV status disclosure, and that the decision to use condoms mostly depended on the willingness of the male partner. Our finding confirmed that husband/partners’ of female migrant workers engaged in condomless sex despite knowing their partner’s positive status. This study suggests that HIV prevention strategies for this population should not be limited to encouraging disclosure but should adopt a more integrated approach including delivery of clear message on the risk of HIV transmission and strategies for HIV prevention in the context of the gender norms and male dominance over women within the Shan community. Lack of knowledge regarding HIV prevention and treatment could be a reason for the husbands/partners not to engage in safe sex practices. While female migrant workers were educated on HIV prevention and treatment, sexual and reproductive health, their husband/partner were less likely to be exposed to such information.

It is likely that Shan female migrant workers have low perceived risk of HIV transmission given that ARTs under the right conditions can reduce the risk of HIV transmission. This reflects the potentially conflicting information provided by healthcare providers. On one hand, condom use and birth control are strongly encouraged to prevent HIV transmission to the husband/partner and child. On the other hand, healthcare providers underscored the low risk of HIV transmission when there is good adherence to ART. For this reason, Shan female migrant workers who did not want to risk disclosing their HIV status by using condoms felt they were not endangering their husbands/partners as long as they maintained good adherence to their HIV medication. The fact that many had children after receiving the HIV diagnosis is another indicator of their low risk perception. Our findings highlight the need for the careful delivery of HIV risk-reduction messages in the context of ART.

Notably, Shan female migrant workers demonstrated a high level of adherence to ART, but also faced specific barriers to this adherence. These included for example high mobility and frequent relocation because of their migrant status. That was compounded by stigmatization of HIV and fear of unwanted disclosure of HIV in their community which caused some of these women to refill ART in distant health facilities, which often resulted in missing clinical appointments due to lack of transportation or money.

HIV infection remains highly stigmatized in many communities. Policies encouraging disclosure of HIV status should be implemented carefully to avoid negative outcomes such as rejection and stigmatization of those infected with HIV. These policies should also be sensitive to socio-cultural and contextual factors, and tailored to the needs of HIV-infected female migrant workers, including their sexual and reproductive needs. Illustratively, the female migrant workers’ desire to have children with their current husbands could be so important to them that they would downplay the potential risk of HIV transmission to the child. Hence, the current policy that encourages birth control to limit mother-to-child HIV transmission in migrant workers might fall short of its aim.

Conducting the interviews in health facilities was one limitation of this study. This setting could have potentially encouraged socially-desirable answers on sensitive issues such as ART adherence, sexual practices and HIV disclosure. The research team may have been viewed as representatives from the public health sector which may have caused participants to inadvertently fear that their responses would affect the healthcare they received. In addition, it is not clear to what extent our findings reflect realities of migrant workers from other ethnic groups in other settings of Thailand. However, the strength of this study is that it triangulated data from the quantitative survey with qualitative interviews to grasp issues around sexual life and disclosure of HIV status among Shan female migrant workers in Chiang Mai.

### Conclusions

This study highlighted the complex challenges of HIV status disclosure among HIV-positive Shan female migrant workers. Fear of marital conflicts and/or fear of loss of financial support from husbands/partners, fueled by the social stigma associated with HIV, prevented female migrant workers from disclosing their HIV status to their husband/partner and network members. Non-disclosure of HIV status negatively impacted their capacity to negotiate for safe sex, but condomless sex even in the context of HIV status disclosure underlined the gender norms and male dominance over women in decision-making for safe sex. Strategies aiming to facilitate disclosure of HIV status and improve condom use among Shan migrant workers should address these socio-cultural barriers. Messages to encourage condom use should be carefully delivered so that information about good ART adherence to reduce HIV transmission does not become a barrier to condom use.

## Supporting information

S1 File IDI guidelines of participants and health providers. (DOCX)
