# Supplementary material for: Disclosure of HIV status among Shan female migrant workers living with HIV in Northern Thailand: A qualitative study
Source: PLoS One. 2019 May 2;14(5):e0216382. doi: 10.1371/journal.pone.0216382 (PMC6497284; doi:10.1371/journal.pone.0216382)
Supplement: S1 File — (DOCX) [file pone.0216382.s001.docx]

**In-depth Interview Guideline**

**For Participant**

Hello. My name is.........(Insert interviewer’s name) ....... I’m a researcher from Research Institute for Health Sciences, Chiang Mai University. Today, as you are healthcare provider, I’d like to interview you for in-depth information on your routine, health behavior, including the health services you’ve used.

You’ve already been explained about the study in our previous discussion. Before we start the interview, do you feel comfortable to proceed or do you have any questions? If you feel comfortable and have no question, I’d like to ask for your permission to record our discussion. You might feel uncomfortable answering some questions or being recorded during the interview. If so, you can choose not to answer the particular question, ask me to stop the recording at some point, or even stop the discussion at any time. Besides, you can withdraw from the study at any time. You don’t need to be in the study throughout the study duration or provide any reason for your withdrawal.

**Turn on the recorder**

This is … (Interviewer’s name) …

Interviewing … (Interviewee’s ID) …

on… date month year) …

**Interview Topics**

- **Participant baseline data** (i.e. personal background, family, work, blood testing history and HIV test result notification)
- **Routine after learning HIV test result**
- Since you’ve learned your test result, how have you adjusted after learning about your HIV infection?
- How did your family members react when they learned about your HIV test result?
- After learning your HIV test result, how do you spend your routine and work?
- How often have you meet intimates, relatives or friends? Did you join community activities? How?
- How were you getting along with other migrant workers?
- How was your relationship with employer/colleagues after they learned about your HIV infection?
- Have you experienced discrimination or rejection from community? How was it? How did you react to the situation?
- How was your sexual practice and condom use after learning about your HIV infection? Could you tell me more about it?
- **Health and self-care**
- Have you taken ARV? Since when? How do you adjust to maintain drug adherence and handle the side effects? What is your understanding about ARV?
- Have you used other alternative medicines (such as herbs, traditional treatment, soul/superstitious treatment and etc.)? How?
- Do you have HIV-related and non-HIV related illnesses? If so, what are they?
- Do you have mental/mental health problem from HIV infection? If so, what are they?
- Do you disclose your HIV status? How and why?
- **Using health services**
- How do you choose HIV-related health services or healthcare facility? Have you changed to the new facility? Why?
- How do you choose non-HIV related health services or healthcare facility?
- Do you have problems or limitations when using the health services at a clinic or hospital? What are they? Could you tell me more about them?
- Have you experienced HIV-related stigmatization or discrimination?
- What kind of health services do you think should be improved? In what aspects?
- Do you think being migrant worker living with HIV make hospital staff treat you differently from others? How? Could you tell me in details or give an example?
- **Life perspectives towards present and future**
- How does living with HIV affect your life?
- What do you think about your life now?
- How do you plan about your future?
- What do you think are the similarities or differences between living in Thailand and in your homeland in terms of way of living, self-caring or receiving health services? Would they be the same or different? How?

**In-depth Interview Guideline**

**For Healthcare Staff**

Hello. My name is..........(Insert interviewer’s name) ............. I’m a researcher from Research Institute for Health Sciences, Chiang Mai University. Today, as you are healthcare provider, I’d like to interview you for in-depth information on health behavior and the use of health service of migrant workers living with HIV.

You’ve already been explained about the study in our previous discussion. Before we start the interview, do you feel comfortable to proceed or do you have any questions? If you feel comfortable and have no question, I’d like to ask for your permission to record our discussion. You might feel uncomfortable answering some questions or being recorded during the interview. If so, you can choose not to answer the particular question, ask me to stop the recording at some point, or even stop the discussion at any time. Besides, you can withdraw from the study at any time. You don’t need to be in the study throughout the study duration or provide any reason for your withdrawal.

**Turn on the recorder**

This is … (Interviewer’s name) …

Interviewing … (Interviewee’s ID) …

on… (date month year) …

**Interview Topics**

- **Participant baseline data** (i.e. personal background, work, details of providing health services for migrant workers living with HIV)
- **Health and self-care of migrant workers living with HIV**
- From your experience working with migrant workers living with HIV, what do you think about their self-care?
- What are general details of their ARV medication?
  - What funds/projects do they receive ARV from?
  - How do they adjust themselves in order to maintain drug adherence or handle the side effects?
  - What is their understanding about ARV?
- Do you know whether they use other alternative medicines (such as herbs, traditional treatment, soul/superstitious treatment and etc.)?
- What HIV related/unrelated illness do they usually have? How much is the chance they get complications from HIV?
- Do you think they have mental/mental health problem from HIV infection? How?
- Do they disclose their HIV status? How and why?
- How much is the chance they transmit HIV virus to their partner or those sharing drug with them?
- **Providing health services**
- In your view, how migrant workers living with HIV choose their health services or health facilities? What are the reasons?
- Do migrant workers living with HIV use non-HIV related health services or choose healthcare facilities? How?
- Do you have any problems or limitations when providing health services to migrant workers living with HIV? If so, what are they? Could you tell me more about them?
- Have you ever witnessed any stigmatization or discrimination against migrant workers? How did it happen in health care facilities or in the community?
- What kind of health services for migrant workers do you think should be improved? In what aspects?
- In general, do you think hospital staff treats migrant workers living with HIV differently from others? How?
